# How is the implementation of empirical research results documented in conflict-affected settings? Findings from a scoping review of peer-reviewed literature

**DOI:** 10.1186/s13031-023-00534-9

**Published:** 2023-08-22

**Authors:** Enrica Leresche, Mazeda Hossain, Maria Livia De Rubeis, Veerle Hermans, Doris Burtscher, Rodolfo Rossi, Cordelia Lonsdale, Neha S. Singh

**Affiliations:** 1 Department of Global Health and Development, London School of Hygiene and Tropical Medicine, London, UK; 2 Global Health, Nottingham Trent University, Nottingham, UK; 3 LuxOR, Médecins Sans Frontières Operational Centre Brussels, Luxembourg, Luxembourg; 4 Médecins Sans Frontières Vienna Evaluation Unit, Vienna, Austria; 5 Centre for Operational Research and Experience (CORE), International Committee of the Red Cross, Geneva, Switzerland; 6 Elrha’s Research for Health in Humanitarian Crises Programme, Cardiff, UK

**Keywords:** Implementation, Humanitarian, Operational research, Knowledge, Normalization Process Theory, Public health, Conflict

## Abstract

Implementation science scholars argue that knowing ‘what works’ in public health is insufficient to change practices, without understanding ‘how’, ‘where’ and ‘why’ something works. In the peer reviewed literature on conflict-affected settings, challenges to produce research, make decisions informed by evidence, or deliver services are documented, but what about the understanding of ‘how’, ‘where’ and ‘why’ changes occur? We explored these questions through a scoping review of peer-reviewed literature based on core dimensions of the Extended Normalization Process Theory. We selected papers that provided data on how something might work (who is involved and how?), where (in what organizational arrangements or contexts?) and why (what was done?). We searched the Global Health, Medline, Embase databases. We screened 2054 abstracts and 128 full texts. We included 22 papers (of which 15 related to mental health interventions) and analysed them thematically. We had the results revised critically by co-authors experienced in operational research in conflict-affected settings. Using an implementation science lens, we found that: (a) implementing actors are often engaged after research is produced to discuss feasibility; (b) new interventions or delivery modalities need to be flexible; (c) disruptions affect how research findings can lead to sustained practices; (d) strong leadership and stable resources are crucial for frontline actors; (e) creating a safe learning space to discuss challenges is difficult; (f) feasibility in such settings needs to be balanced. Lastly, communities and frontline actors need to be engaged as early as possible in the research process. We used our findings to adapt the Extended Normalization Process Theory for operational research in settings affected by conflicts. Other theories used by researchers to document the implementation processes need to be studied further.

**Supplementary Information:**

The online version contains supplementary material available at 10.1186/s13031-023-00534-9.

## Background

Conducting operational research in conflict-affected settings is limited by insecurity, inconsistent data, ethical issues, political instabilities, populations movements and fragmented governance [1–4]. A persisting research gap has been documented in such settings over the past two decades [5–8]. To fill this gap, efforts have focused on producing more high-quality research [9–12], recognizing that subsequent decision-making for policy and planning depends on the quality and relevance of the evidence base, on the type of organizational support, and on the level of disruptions [13–16]. But once operational research is conducted and decisions are made to change policies or plans, does it follow that practices will be adjusted? This assumption is questioned by scholars working on the implementation of evidence-based public health practices in more stable settings [17–20]. They found that knowing ‘what works*’* is not enough; changing practices means considering implementation as a social process, engaging practitioners to learn dynamically, and looking at system complexities [18, 21, 22].

In conflict-affected settings, services delivery is known to be limited by violence, insecurity, logistical constraints, delivery gaps, inflexible funding, weak coordination, and fragmented leadership [23–27]. Understanding how these limitations can be overcome and by whom is unclear. Grasping how empirical results might lead to revised practices is complex in conflict-affected settings [28–30]. This paper applied an implementation science lens to analyse how scholars documented this process in the peer-reviewed literature.

Within implementation science, ‘*who is involved?*’, *‘to do what?*’ and ‘*where?*’ are core questions used to identify implementation mechanisms [19, 20]. We chose the Extended Normalization Process Theory (ENPT) because it focuses on implementing actors’ agency and questions the capacities and the potential of actors [18, 31]. Given recognized power imbalances in humanitarian settings, choosing a framework that accounts for the agency of frontline actors was important [1, 32]. In a previous study we found that using the ENPT allowed one to analyse how actors negotiated constraints collectively [33]. Our results also generated further questions to be assessed in the peer-reviewed literature:

1. How are implementing actors engaged by researchers to produce knowledge?
2. Do the characteristics of the intervention matter?
3. How do contextual and organizational factors influence implementing actors?
4. What role do implementation actors play?
5. What does implementing revised practices look like?
6. How do actors negotiate the implementation process?

In this review we assessed how authors documented how new empirical research results were integrated into routine practices by implementing actors (used interchangeably with frontline actor in this paper) in settings affected by conflict or violence.

## Methods

We conducted a scoping review of the peer-reviewed literature to understand better how scholars documented and understood the process by which empirical research findings lead to revised practices in conflict-affected settings. We based our work on implementation science tools and established criteria to include papers that described the interaction between actors, contexts, and new interventions. The data extraction and analysis were deductive when assessing how intervention characteristics and contextual factors emerged in our data based on known implementation science concepts. An inductive analysis was used to analyse data on the roles that actors played, what implementation looked like and what was negotiated, in order to use our results to adapt the ENPT further. Both steps allowed us to respond to the six questions presented in the background section. The first three questions are based on existing implementation science concepts and were used for the deductive data analysis. Responses to questions four to six were used for our inductive analysis, to adapt and develop the dimensions of the ENPT for operational research in settings affected by conflict.

We narrowed the scope to empirical results that suggested changes (as opposed to usual policies or prevailing practices) to focus on how new knowledge is integrated into practices, given that humanitarian organizations are known to struggle to learn and integrate changes [34, 35]. Excluding interventions known to be effective also aimed to assess how implementing actors relate to knowledge production (in the context, for example, of operational research or embedded research) in conflict affected settings. In included papers we identified ‘*how*’ specific barriers were overcome and ‘*who*’ contributed to the change.

### Inclusion and exclusion criteria

To be eligible, papers had to (a) consider the actors, the interventions and the contexts, to present data on key dimensions (the context, the actors, the intervention) described by implementation science scholars [18, 20, 30]. In addition, papers had to (b) focus on a public health intervention implemented by a humanitarian organisation; (c) be peer-reviewed; (d) consider new empirical results; (e) occur in settings affected by conflict or political violence; (f) and exclude clinical procedures or military medicine. All study designs were considered, and interventions had to be based on previous research conducted. These criteria were used to assess how changes were initiated from research to humanitarian practice. We focused on conflict-affected settings to explore the combined effect of political violence, population movements, and instability on research to practice processes. We excluded clinical procedures or military medicine because they take place in more controlled environments, whereas our aim was to understand better the challenges of implementing interventions within communities. The quality of the initial research was analysed descriptively. The documentation process was assessed based on criteria for quality implementation research [36]. The search including records in English from year 2000 to November 2021. This 20-year time frame corresponds to the global recognition that humanitarian responses need to be more systematically based on empirical research results [5, 7, 14, 37, 38]. We used specific definitions in this paper (Table 1).

**Table 1:** Definitions used in this paper

	Definitions used in this paper	Citation(s) that the definition is based on
Broader health system	Includes the actors, resources, systems, policies, and processes related to the provision of healthcare in each setting	Authors
Core components of an intervention	The main and essential elements that compose an intervention and are understood to be needed for it to work as expected	[18, 39]
Implementation of revised practices	The individual or collective set of efforts and actions to bring research recommendations into routinely revised practices for public health	[17, 40]
Frontline actor (s)	Individuals and groups that encounter each other in health care settings to provide care, including community health workers or lay providers. Used interchangeably with implementing actor	[41, 42]
New empirical findings	Research results that bring a new form of knowledge and imply a change in practice	Authors
Peripheral component of an intervention	A component that is not the core of the intervention and can be modified, for example the modalities through which an intervention is delivered	[18]
Scientific knowledge	Used interchangeably with empirically validated research results. Findings resulting from a scientific and empirical process including qualitative and quantitative methods	Authors

### Information sources

Three public health databases were searched: ‘*Global Health*’, ‘*Medline*’, and ‘*Embase’,* using the Ovid interface. The search strategy was built with a senior librarian from the London School of Hygiene and Tropical Medicine (LSHTM). Search terms were combined into three main concepts: ‘*Empirical research production, use, or implementation’* AND ‘*Evidence-based practices’* AND ‘*humanitarian contexts’*. This strategy allowed us to include new empirical findings and their use and implementation in practice, in conflict-affected settings based on core implementation concepts. Key words and subject headings were explored for each concept and combined with Boolean operators. Subject headings were adapted to each database. An example of the search strategy and search terms is available (Additional file 1). 

### Selection process

Abstracts and full-text screening was conducted by EL. Any papers with unclear eligibility were discussed with NSS and MH to reach an agreement.

### Data extraction

Informed by core concepts in implementation science, we developed a data extraction table, based on the following questions: What was the study design? What was the intervention? How was knowledge produced? How was the research process organised? What were the characteristics of the intervention? What were the internal organizational structural capacities? How was the external context approached? How was the relationship with humanitarian actors described? How was the relationship with communities approached? What was negotiated?

### Data synthesis, and critical analysis by co-authors

The responses to these questions allowed us to analyse how the implementation of new knowledge was documented in papers selected. All themes were identified in the body of each paper using a narrative synthesis [43]. The data were extracted and analyzed by EL and discussed with NS and MH. Once the key results were synthetized, a group of actors involved in framing, sharing, using or discussing operational research results with field teams from MSF, ICRC and Elrha (MLR, VH, DB, RR and CL) provided critical feedback and questioned the results, proposed additional syntheses to be made, or literature to be integrated into the discussion section. That step allowed us to review our results based on the perspective from actors involved in operational research in conflict-affected settings.

### Ethics

This research project was approved on 14 December 2021 by the ICRC (*2118_Nov* DP_DIR 21/00031 CGB/bap), on 21 December 2021 by the LSHTM (*Ref.26482*) and on 11 April 2022 by Médecins-Sans-Frontières (MSF) (*ID 2177*).

## Results

**Fig. 1:**
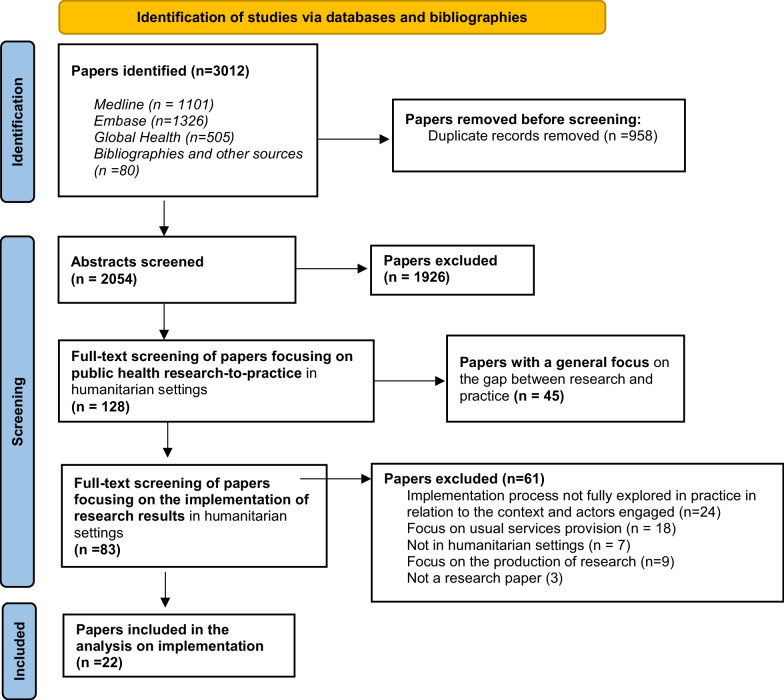
PRISMA flowchart

A total of 2054 abstracts were screened. After full-text review, 22 papers met the inclusion criteria (Fig. 1).

Table 2 presents a summary of all papers included (n = 22), published between 2011 and 2021. Two-thirds describe the implementation of mental health interventions (n = 15/22). Geographical contexts include 34 countries (Asia (n = 11), the Middle East (n = 4); Africa (n = 13) and South America (n = 6), either experiencing conflicts, affected by conflict, or recovering from conflict or violence.

**Table 2:** Papers included in the scoping review on the implementation of research findings in humanitarian practices

Authors	References	Intervention	Evidence bases for the initial intervention proposed	Study design, aim & methods to assess the implementation process	Context (s)
Bennett et al. (2017)	[44]	Quality of care score card Community Health & telemedicine Maternal and neonatal health	Conducted implementation research Conducted household surveys Conducted focus group discussions Analysed quarterly longitudinal data	Retrospective To identify scale-up strategies Documentary analysis & field teams consultations	Afghanistan, Bangladesh, Uganda
Paina et al. (2017)	[45]			Retrospective To assess modifications of theory of changes Documentary analysis & field teams consultations	Bangladesh, India Uganda
Bosqui et al. (2020)	[48]	Psychoeducational comic book	Cites systematic reviews Conducted a systematic review	Prospective To use review findings to develop a book Pilot dissemination, qualitative interviews	Lebanon
Brown et al. (2020)	[60]	Early Adolescent Skills for Emotions (EASE)	WHO guidance Cites a systematic review	Retrospective To adapt and implement an intervention Cognitive interviews, workshops, scoping review	Lebanon
Foster et al. (2017)	[50]	Community-based distribution of Misoprostol	WHO guidance Cites a single arm prospective study Cites a literature review of controlled trials	Retrospective To measure pregnancy outcomes Monitoring logbooks & open-ended interviews	Thailand-Myanmar border
Fuhr et al. (2020)	[54]	Problem Management Plus (PM +)	WHO guidance Cites a single-blind RCT	Prospective planning To test the Theory of Change pathways to scaling up Participative workshop, review with peers	Turkey
Fuhr et al. (2020)	[55]	Problem Management Plus Early Adolescent Skills for Emotions	Cites a single-blind RCT WHO guidance	Prospective planning To elaborate pathways to changes across countries Systematic review, short appraisal, workshops	Turkey, Lebanon, and Netherlands
Giordano et al. (2021)	[64]	Mental health and Psychosocial	Cites field research Cites a literature review Conducted field research	Retrospective To propose a ladder of program adaptation Literature review and case studies	Humanitarian settings
Kienzler et al. (2019)	[65]	Mental health systems reforms	Conducted field research Conducted case studies	Retrospective To document what actors do in practice Ethnographic case-studies and literature review	Kosovo, Palestine
Jordans et al. (2011)	[47]	Psychosocial care package for children Multi-layered psychosocial care package	Conducted a systematic literature review and narrative literature review Technical experts and practitioners consultations	Prospective pilot To develop a strategy to select a feasible treatment Focus group discussions, semi-structured and in-depth interviews, literature reviews, expert panel	Burundi
Jordans et al. (2011)	[59]	Psychosocial care package for children, Multi-layered psychosocial care package	All of the work described in [47, 59] WHO guidance Cites a review Conducts an applied evaluation	Retrospective practice-driven To assess patient and provider perceptions, satisfaction, burden, access, and cost Monitoring data, questionnaires to providers and beneficiaries, qualitative data, and cost analysis	Burundi, Sudan, Sri Lanka, and Indonesia
Jordans et al. (2013)	[49]	Psychosocial care package for children Multi-layered psychosocial care package	All of the work described in [53] Conducts a practice-driven evaluation Conducts a pilot test	Retrospective case studies To address challenges (for processes & outcomes) Document review, cluster randomized trials, qualitative research, resources analysis	Burundi, Sudan, Sri Lanka, and Indonesia
Schauer et al. (2017)	[53]	Micronutrient Powders (MNP) interventions	WHO guidance	Retrospective Document 1) planning and supply; 2) behaviour change and training; and 3) monitoring & evaluation Literature reviews, synthesis of programmatic documents, working groups & key informant interviews	Post-conflict countries in Africa, Asia, and South America
Reerink et al. (2017)	[52]	Micronutrient Powders (MNP) interventions	WHO guidance Literature review (randomized trials)		Post-conflict countries in Africa, Asia and South America
Vossenaar et al. (2017)	[51]	Micronutrient Powders (MNP) interventions	WHO guidance Literature review (randomized trials)		Post-conflict countries in Africa, Asia and South America
Sandvik et al. (2013)	[46]	Right to food aid Status of IDP women	Based on a legal requirement	Retrospective ethnographic case study Examine the enjoyment of rights in persisting conflict Interviews, field review, practitioner feedback	Colombia
Sangrula et al. (2021)	[58]	Problem Management Plus	Conducted a feasibility study WHO guidance	Retrospective, part of a clinical trial project To propose a methodology to assess implementation Systematic literature review, qualitative interviews	Nepal
Miller et al. (2016)	[61]	Model for children’s mental health	Cites systematic reviews Cites an RCT	Retrospective To compare literature review with interventions Review of documented practices	Conflict affected settings
Murray et al. (2014)	[57]	Evidence-based mental health interventions	WHO guidance	Retrospective To outline key implementation challenges Experts collective experiences and literature review	DRC, Iraq, Colombia, Thailand-Myanmar border
Nemiro et al. (2021)	[56]	Problem Management Plus	Cites RCTs WHO guidance	Retrospective case studies To evaluate the implementation outcomes Document review and semi-structured interviews	Ethiopia, Syria, Honduras
Perera et al. (2020)	[62]	Problem Management Plus	Cites a systematic review Cites field research Refers to an RCT	Retrospective Adapt an intervention and develop guidance Literature review; qualitative interviews; expert panel	Colombia
Wieling et al. (2015)	[63]	Enhancing Family Connections (EFC)	Cites a randomized experimental study Refers to a randomized trial	Retrospective To document adaptation & implementation Literature review, focus group discussions, mother–child interviews, ethnographic approach	Northern Uganda

To build the *evidence base for new interventions (by methodology)* several authors conducted primary research including household surveys [44–46]; qualitative studies [44, 45]; systematic literature reviews [47–49]; or retrospective case studies [49]. Authors also referenced existing evidence including WHO guidance [50–60]; prospective studies [50]; RCTs [54–57, 61, 62]; randomized trials [63]; surveys [51], operational research [44–46, 49, 58, 64, 65]; systematic literature reviews [48, 60–62], and literature reviews [47, 50–52, 59, 64].

More specifically, the type of *expected benefits* for each intervention are presented in Table 3. Most authors (n = 14) anticipated a benefit based on external findings from systematic reviews [66, 67], WHO guidance [68–73], randomized trials [74, 75], cross-sectional surveys or cohort studies [76–78], or a literature review [79]. Other authors either drew from mixed approaches (n = 4) or expect benefits from their own research based on case studies, monitoring results, or qualitative work (n = 4).

**Table 3:** Expected benefits from each intervention and source of information

	Mainly based on own empirical field research derived from practice (n = 4)	Mainly based on external evidence referenced (n = 14)	Mixed approach (n = 4)
Authors using this approach	[44–46, 65]	[48, 50–58, 60–63]	[47, 49, 59, 64]
Type of intervention, benefits described, and references used to describe these benefits	*° Community scorecards* are expected to be a means to engage multiple stakeholders, strengthen social accountability and responsiveness [44] *° Functional call centres* are expected to increase the support for, and quality of care provided by informal healthcare providers [44] *° Multifaceted mother and child interventions* are expected to stimulate the demand for services (through transport vouchers and sensitization) and improve the quality of services (through trainings, non-financial recognition, supportive supervision) [44] *° Practice-based mental health interventions* are expected to bring longer term changes in the health system [80] *° Implementation of government policies* are expected to decrease food insecurity [46]	*° Self-guided help* is expected to improve mental health outcomes [66, 67] *° EASE* is expected to help adolescents cope with depression or anxiety symptoms [69] *° Misoprostol* is expected to be between 75 and 90% effective to induce abortion in the first 9 weeks of pregnancy [76, 79] *° PM*+ is expected to reduce common mental health symptoms and improve psychosocial functioning [68, 70, 74] *° MNP* is expected to decrease and prevent micronutrient deficiencies [71] *° Social factors* are expected to influence the well-being of children [77, 78] *° Evidence-based mental healthcare* interventions improve mental health outcomes [72, 73] *° Evidence on Parenting interventions* are expected to increase the well-being of children [75]	*° Developing a tutor of resilience approach* is expected to increase the feasibility and sustainability of the intervention [64] *° Inclusion of families* in the model is expected to increase the resilience of the children [47, 77, 81] *° Multi-layered mental health programs* are expected to be more effective [59, 82, 83] *° A system of care approach tailored to the context* is expected to increase effectiveness [49, 84, 85]

*To assess and document the implementation of these new interventions*, study designs were mostly qualitative (n = 21) (e.g., field team consultations, focus group discussions, ethnographic case studies). Several studies used retrospective records reviews (n = 9) or studied monitoring data (n = 10). One study used a cluster randomised trial and two included a costing analysis. Mostly mixed methods approaches were used to assess the implementation process.

### How are implementing actors engaged to produce new knowledge?

Table 4 presents the use of implementing actors’ knowledge, categorized across a continuum of four levels [64, 65, 86]. Most research was described as initiated by external academics [49–53, 56, 57, 61, 64] and later shifted to collaborative strategies [48, 51–55, 58–60]. Sometimes implementing actors conducted research [44–47, 64] but more often were engaged afterwards [49–56, 58, 59]. Sometimes, the entire continuum was covered across multiple projects [64]; or in different settings and over time [47, 49, 51, 52, 59]. Papers covering less than a one-year time frame and in a restricted number of settings often touched upon a more limited set of components [47, 54, 57, 58, 61, 64, 87].

**Table 4:** The connection between knowledge production and implementing actors in a continuum

Knowledge production was initially detached from implementing actor’s practice	Knowledge was adapted through an interface with implementing actors	Knowledge emerges from a dynamic and interactive learning space	Knowledge emerges from the practitioners and from communities themselves
(1) ⟶	(2) ⟶	(3) ⟶	(4)
° Experts developed an intervention, notion of rigorous methodologies, established global evidence, strong scientific validity [49–53, 56, 57, 61, 64] ° Mention the gap between research and practice, and the need to understand how to implement or scale up effective interventions [51–54]	° Piloting, monitor, evaluate and adapt interventions [48, 51–55, 58–60] ° Address poor translation of knowledge into practice [48, 57] ° Mixed approaches (epidemiology, economics, social sciences) to document changes and adapt throughout the process [51–53, 57–59] ° Establish protocols, resources centres, ensure fidelity [49, 51–53, 55, 56, 59]	° Interactive learning spaces are open over time [44, 45, 49, 63] ° Participatory learning processes, co-thinking, co-production of knowledge [44–46] ° Shared responsibilities [44, 62, 63, 65] ° Implementing actor bring knowledge on practice and context [47, 49, 51–54, 58, 59] ° Discuss findings with communities and practitioners [44–47, 54, 63, 65] ° Notion of learning from practice [49, 51–53, 58, 63]	° The core notion of evidence is re-examined [46, 49, 63, 65] ° Knowledge is power, represents a cultural capital [46, 48] ° Unintended negative impact when interventions are imported [64] ° Need to decolonialise knowledge [64] ° Practices produce knowledge [49, 65]

### Do the characteristics of the interventions matter?

Key characteristics of new interventions (focus, flexibility, main user) are described in Table 5. Most papers (n = 15) evaluated a Mental Health and Psychosocial support (MHPSS) intervention. Other interventions (n = 7) related to general service delivery, community health, nutrition, or Maternal and Child Health (MCH). In most cases, implementation meant that the core mechanism of interventions remained unchanged, but modalities such as mobile strategies or packages were adapted*.* Few papers described an in-depth adaptation of the intervention [44, 45, 64]. The level of flexibility and adaptability were reported as key to successful implementation [44, 47–49, 56, 58, 63]. Adapting also meant balancing efficacy or effectiveness with what would be feasible, acceptable, or culturally valid [44, 49, 51–53, 55, 58, 62, 63]. For interventions that were less adaptable, such as the clinical provision of Misoprostol [50], or micronutrient powder (MNP) [51–53], discussing delivery strategies was essential. When services providers co-produced interventions with researchers, such as scorecards [44], local communities were engaged earlier in the adaptation process.

**Table 5:** Characteristics of the interventions implemented

Focus of the intervention	Flexibility	Intervention implemented by
° Services delivery and quality of the services (score card, services referrals) [44, 45] ° Maternal and Child Health (Misoprostol, care package) [44, 45, 50] ° Community health tools (telemedicine and health box) [44, 45] ° Nutrition or food assistance (Micronutrient powders and food support) [46, 51–53] ° Mental health and psychosocial support (PM+, EASE, Somoud comic book, parenting intervention, tiered care package, self-help approach) [47, 48, 54, 55, 57, 59, 61, 63, 64]	° Flexible core: intervention itself can be modified in-depth [44, 45, 64] ° Flexible periphery: strategies or delivery options: early adaptations, components can be added or suppressed from the intervention itself [47, 48, 54–59, 63, 64] ° Standard and mostly non-flexible intervention itself but delivery strategies can be adapted [50–53]	° Lay community providers [44, 47, 50, 52, 54, 55, 58, 59, 64] ° Skilled services providers [44, 48, 52–55, 57, 59, 63, 64] ° Researchers for advocacy [46, 65]

### How do external and organisational factors affect implementing actors?

#### External factors

External factors created disruptions (Table 6). In order to cope with disrupted social, political, or cultural environments implementing actors adapted continually to instability, violence and changing interests [44, 45, 47, 49, 52, 53, 57–59, 61, 62, 64]. Disrupted economies meant that actors constantly negotiated resources [44, 47, 52, 53, 63]. Precarious leadership and weak or unstable policies led to unsteady engagements [52, 54, 55, 57, 64], and difficulties to elaborate longer term plans [44, 45, 52–55, 59]. Political disruptions meant that research neutrality was questioned, and politicized interests needed to be considered [44, 54]. Limited human resources and irregular access interrupted changes in practices [44, 49, 53–55, 57, 59]. Populations sometimes did not trust understaffed or politicized services, and restoring trust was challenging [44, 47, 50, 57, 58]. Security and legal constraints were difficult to solve, limiting access to existing services [44, 50, 62] or the right to work [54, 55, 62].

**Table 6:** Main conflict-related disruptions affecting the implementation of new practices

Overall environment	Health system	Security and legal issues
° Disrupted social, political, geographic, cultural environments and violence [44, 45, 47, 49, 52, 53, 57–59, 61, 62, 64] ° Disrupted economies and poverty [44, 47, 52, 53, 63] ° Instable network of stakeholders [52, 54, 55, 57, 64] ° Unsteady leadership, governance, and policies [44, 45, 47, 49, 52–55]	° Multiple layers and levels are affected [45, 47, 49, 52–55, 59] ° Insufficient or disrupted resources [44, 49, 53–55, 57, 59] ° Stigma or lack of trust [47, 50, 57, 58] ° Inflexible funding or funds channelled to specialized care [54, 55, 57]	° Legal restrictions for people affected [44, 50, 62] ° Restricted legal environment for services providers [54, 55, 62]

#### Organizational factors

Organizational factors were documented as influencing the capacity to implement new practices (Table 7). More specifically, the capacity to increase, recruit or mobilise human resources was crucial [44, 46, 47, 49, 50, 52–56, 59, 60, 62, 63]. Task shifting sometimes allowed implementers to cope with shortages of qualified staff or achieved improved community uptake [53–55, 57]. The need for additional training was widely documented [44, 46, 49, 51, 52, 56, 58, 59, 61, 63], while ensuring that newly trained staff had sufficient time for additional tasks [51, 52, 54]. Implementing a change in practice was also influenced by the learning environment*.* Interactive tools facilitated the link between research and ongoing operations [44, 45, 49, 53]. Sometimes technical committees were set up to discuss implementation processes [45, 47, 49, 51]. When actors interacted frequently with external stakeholders, the process was easier [44, 50, 52–54]. Embedding new interventions in existing programs also increased the capacity of actors to implement changes to ongoing practices [44, 45, 48, 49, 52, 63].

**Table 7:** Organizational factors affecting the learning environment

Resources allocated	Learning environment	Continuity & coordination
° Increase, recruit, or mobilise human resources [44, 46, 47, 49, 50, 52–56, 59, 60, 62, 63] ° Task shifting [50, 53–55, 57, 59] ° Training facilitators and community counsellors [44, 46, 49, 51, 52, 56, 58, 59, 61, 63] ° Build cultural and communication skills actors [52, 61–63]	° Interactive tools [44, 45, 49, 53, 58] ° Technical groups [44, 45, 47, 49, 51, 54, 55, 62] ° Supportive supervisions [44, 45, 49, 54, 55, 63, 88] ° Use the monitoring system for critical thinking [45, 49–51, 56] ° Consider language [56, 58, 62–64]	° Capacity to connect inside and outside [44, 50, 52–54] ° Connections across sectors [45, 51–53, 56] ° Build on previous experience [44, 46, 48, 49, 52, 56, 59, 63, 65] ° Allocate time [49, 51–53, 56, 58, 63, 65]

### What roles do implementation actors play

Implementing actors included national or international services providers or academics (training services providers or adapting interventions) [44, 45, 47–59, 64, 65]. Services providers embedded in communities were perceived to be more trusted [47, 48, 50, 56, 58, 63]. Sharing new knowledge mostly consisted in trainings, followed by supervisions and technical support [44, 49–52, 56–59, 61–63]. Service providers discussed acceptability, cultural validity, acceptability, and negotiated feasibility [47, 49, 52, 56, 58, 63, 64, 89]. They were keen to have intense learning interactions [44–46, 64] especially if they had contributed to the design of the research [47, 49, 51–53, 58, 59, 64]. Service providers often experienced heavy workloads, facing complex logistical demands, had unmet training needs, and experienced attrition of personnel and incessant turnover [46, 51, 52, 57, 59, 63]. Despite such stressors, they led implementation, identified services users, shaped interventions, and raised community awareness [44–47, 49–51, 56, 58, 65].

Implementing actors also included communities that were affected by violence [49, 55; 61, 63]; disrupted social networks [47, 63]; poverty [46, 54, 55, 61, 63]; lack of access to care [51, 57]; fear of stigma [54, 59]; illiteracy [54, 63] and the lack of awareness of service availability [51, 55, 59]. Supportive communities were a key enabler of revised practices as mediated by acceptability, trust, or intention to adopt [45–60, 62–65]. Communities sometimes adapted delivery strategies [44, 45, 47, 58]; used the findings for themselves [46, 64]; produced and managed research findings [46, 64, 65]; or advanced their own rights when political spaces were created [46].

### What does the implementation of revised practices look like?

Implementing recommendations in practice involved negotiating acceptability, appropriateness, or adaptations at the level of the community [44, 47–53, 56, 58, 60, 61, 63–65], often collectively [44, 45, 49, 60, 64]. Assessing adoption, adherence, and fidelity was done alongside concern about cost and feasibility [44, 50, 52, 53, 56, 60, 62, 63]. Implementing adapted practices within a health system meant training, technical assistance, supervision, and logistical support [44, 46, 49–52, 55]. Modifying practices meant getting feedback on progress through existing monitoring tools [49–51, 55, 59]. Implementation outcomes included perceived benefit, effectiveness, coverage, and sustainability [44, 49–53, 57, 63] or, being relevant and meeting unmet needs [44–55, 58, 61, 63]. A detailed measure of cost-effectiveness was rare and only two research papers incorporated cost [49, 52]. Measuring costs was challenging because of the complexity of multiple and changing costs over time [52, 53, 59]. The costing studies were often added at the end of the research process, rather than integrated from the start.

### How do actors negotiate in the implementation process?

Authors documented a range of tensions that needed to be resolved. First, the roles of researchers, stakeholders, services providers, and communities overlapped to negotiate appropriateness and feasibility [47–59, 62, 63]. Second, research recommendations were adapted following unfounded assumptions about partners or health system capacities [45, 51]; new evidence [45]; political, social or cultural changes [45, 48, 51, 58]; the need to prioritize [51, 59]; and feedback from practice [44, 47, 49, 58, 59]. Specifically, balancing fidelity vs. fit was crucial—this can also be described as efficacy vs. feasibility or core vs. peripheral changes. Trade-offs included dropping fidelity for increased adoption [44, 51, 59]. Field supervisions increased fidelity [51, 56] but raised costs, time, and resources [54, 55, 59]. Third, long term resources were negotiated constantly, through funding mechanisms [52]; prioritization [59]; or integration of health interventions in non-health programs [51–53, 87]. Measuring cost-effectiveness was central to sustainability, but was rare, incomplete, or disrupted [52, 59]. Fourth, a balance had to be found between the power of knowledge and the power of practices. Participatory engagements stimulated changes [44–46, 64, 65], while top-down accountability mechanisms were inefficient if social, political or practical constraints were ignored [45, 54, 55]. The power of knowledge sometimes was referred to as a cultural capital to challenge existing power structures or foreign research agendas [46, 48]. The power of practices included communities refusing to lose the benefits of a face-to-face consultation [45] or providers asking for different supports [56]. Sometimes researchers pushed back on suggestions to change core mechanisms of action (expected to modify the main mechanisms of the intervention)[58].

### Contribution of our results to ENPT

**Fig. 2:**
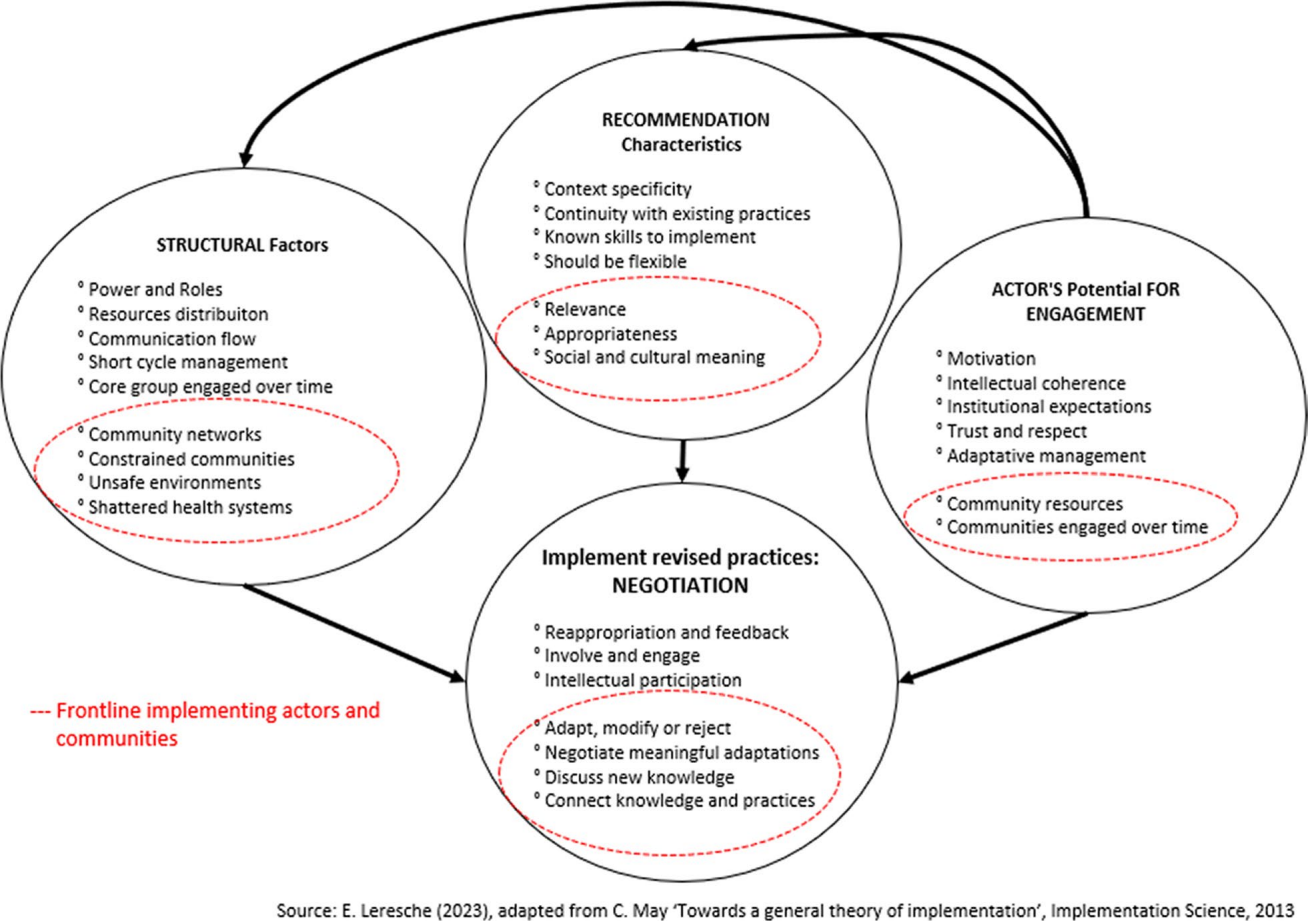
Adapted ENPT (a-ENPT) for the implementation of operational research findings in conflict-affected settings

We gathered data on the description of contextual issues, recommendations characteristics, and the roles that actors played. We observed that in conflict-affected settings, frontline actors faced disruptions (financial, human resources, logistics, security, turnover) that needed specific attention. We also found that new interventions were adapted by frontline actors and communities from different cultural, social, or political backgrounds in tense environments. We found that services providers and communities played an important role not only to adapt recommendations but also to compensate for disruptions (such as lack of staff, or irregular funds). Authors documented that frontline actors negotiated resources, adapted interventions and engaged within communities over time, actively, and in all dimensions of the implementation process and these are important manifestations of their agency (Fig. 2).

Carl May’s ENPT (2013) was used as the basis to propose an adapted ENPT (a-ENPT). Findings from the review were used to refine the theory to reflect on the influence of frontline actors and communities described by authors documenting the implementation process in conflict-affected settings.

## Discussion

We analysed 22 peer-reviewed papers documenting and providing data on implementation processes in settings affected by conflict published between 2011 and 2021. Whether this 10-year timeframe relates to a concurrent increase in awareness of, and guidance on implementation issues globally, would need further inquiry [36, 90].

In these 22 papers we looked at how the interplay between actors, interventions, and contexts was described and conceived. This study did not aim to consider all implementation barriers for interventions that are known to be cost-effective in such settings. Our objective was to analyse papers providing data on processes described by implementation science scholars, and to explore how shaping new practices based on operational research was understood.

We found that most papers considered interventions focused on mental health, that conflict-affected settings presented specific constraints, and that authors documented that frontline actors and communities played a central role in negotiating revised practices.

Most papers in our review focused on mental health. This could be a consequence of our criteria to select papers providing insights on implementation processes, or because mental health scholars may be more accustomed to engaging with implementation science, given that related communities of practice have developed such tools in more stable settings [91, 92]. The focus on mental health interventions could also be a consequence of the increase in research papers and the burden of mental health in conflict-affected settings [93–95]. Despite the focus on mental health, we believe our results have broader utility. Firstly, we considered general characteristics such as adaptability and flexibility. Secondly, engaging implementing actors to negotiate contextual constraints and adapt interventions always mattered. Non-mental health interventions do share such strategies to transform new knowledge into practices. For example, adapting delivery strategies and engaging frontline actors were key to the uptake of MNP or Misoprostol [50–53].

We also found that conflict affected settings involve specific interactions between actors, interventions, and contexts. Frontline actors were displaced or worked temporarily, which altered how they engaged in implementation. Compared to environmental disasters, implementing actors in settings affected by conflict may suffer from insecurity or distrust researchers [96–99]. Engaging frontline actors and communities to develop a relevant research question and feasible recommendations is challenging [100, 101]. Disruptive recommendations are hard to integrate when people are displaced, when human resources turnover is widespread and when organizations struggle to maintain continuity. Humanitarian organizations possibly lack technical research skills or are reluctant to be scrutinized in a competitive system [102, 103]. Measuring financial costs also might not reflect the actual economic cost for communities or total program costs for all stakeholders [104, 105]. These factors are difficult to change. But flexible and stepped approaches, balanced partnerships, progressive task-shifting, interactive supervisions, support centres and learning committees, as well as a respectful and trustworthy engagement over time might be useful [36, 103]. These strategies emerge clearly in our results.

We looked at how implementing new practices was documented and conceived. Peer-reviewed literature documents how to better produce new knowledge [2, 7, 8, 106, 107], on how to use results for policies [14, 16, 108–110], and on existing barriers to services delivery [4, 25, 111–116]. Tools such as the ‘*humanitarian lives saved’* or ‘*uptake guidance’* facilitate decision-making [117–119]. But understanding what these findings and decisions then mean for frontline actors and communities practices in settings affected by conflict, is a yet a different question [15, 103, 120]. Key initiatives reported in the grey literature recognize that supports, trainings, inclusiveness, tailoring research outputs, long term relationships and engaging implementing actors through collective actions are key [103, 119–124]. The necessity to address power imbalances and issues of relevance are also observed [102, 103, 124]. Our focus on peer-reviewed papers is based on the argument that peer-review is expected to increase quality, therefore it is a crucial process to establish an evidence base and to document how changes might occur in practice. Our results showed that sharing an understanding of how empirical research results may lead to revised practices in conflict-affected settings is scarce in peer-reviewed literature and may need to be developed further.

Implementation science scholars advance that knowing *‘*what works*’* alone is not sufficient to bring about changes in practices [19, 20, 30]. The a-ENPT more specifically considers the actors implementing and their potential to engage collectively, which may be crucial in environments where power differentials are known to be exacerbated [1, 32]. In settings affected by conflict, frontline actors and community health workers may build trust, negotiate political sensitivities, or develop sustainable mechanisms [26, 27, 112, 115, 125, 126]. In the set of papers that we analysed, implementing actors reconciled different types of knowledge. The capacity to connect knowledge to practice also echoes the notion of ‘knowledge brokers*’* [39, 103, 127]. Such a role might be possible only if there is enough space and time to negotiate at different levels.

### Strengths and limitations

The main strength of this paper is that it offers an in-depth analysis of how authors described and understood how new knowledge may bring changes in practices in conflict-affected settings, using and adapting an existing implementation science tool.

However, this paper carries a number of limitations. First, the screening of abstracts and data extraction was performed by only one person, though this was compensated for by a thorough discussion of the inclusion criteria and early results with two co-authors (NS, MH). Second, included studies were limited to exclusively peer-reviewed papers that provided data on how the actors interact with the context and the intervention to implement revised practices in settings affected by conflict. While this criterion was necessary to collect data related to implementation science concepts, it clearly considerably limited the number and scope of papers included.

This paper focused on peer reviewed literature. However, crucial work has been conducted by organizations such as the World Health Organization or other organizations that is only available in the grey literature [36, 103, 120, 124]. The recognition that the vast majority of the data relevant to the analysis may be situated in grey literature is an important limitation of this paper. Whether grey literature systematically provides a different perspective on mechanisms by which operational research results lead to revised practices in conflict-affected settings would need to be researched further.

## Conclusion

Our analysis found that implementation actors may negotiate constraints and revise their practices based on new knowledge in conflict affected settings, provided they have the space and flexibility to do so. This study suggests that implementation science perspectives might be useful in settings affected by conflict. Specifically, implementing new practices based on empirical results is not a linear process, whereby providers and communities are passive recipients after decisions are made. Space for negotiation might be needed to debate and challenge recommendations made. This study showed that when authors reflected on the implementation process, actors from within the community or working close to communities need to be engaged early. How such a negotiation can be framed theoretically, and whether the ENPT is the right tool to do so, needs to be researched further.

### Supplementary Information

**Additional file 1**. SEARCH strategy (in this case for Embase).

## Data Availability

The datasets used and/or analysed during the current study are available from the corresponding author on reasonable request.

## Abbreviations

ICRC: International Committee of the Red Cross
IDPs: Internally Displaced Populations
a-ENPT: Adapted Extended Normalization Process Theory
ENPT: Extended Normalization Process Theory
ELRHA: Enhancing Learning and Research in Humanitarian Assistance
LSHTM: London School of Hygiene and Tropical Medicine
MHPSS: Mental Health and Psycho-Social
MSF-OCB: Médecins Sans Frontières Operational Centre Belgium
MCH: Mother and Child Health
MNP: Micronutrient Powder
NGO: Non-Governmental Organization
PRISMA: Preferred Reporting Items for Systematic Reviews and Meta-Analysis
RCTs: Randomized Controlled Trials
