# Supplementary material for: How is the implementation of empirical research results documented in conflict-affected settings? Findings from a scoping review of peer-reviewed literature
Source: Confl Health. 2023 Aug 22;17:39. doi: 10.1186/s13031-023-00534-9 (PMC10464477; doi:10.1186/s13031-023-00534-9)
Supplement: Supplementary file 1 — Additional file 1. SEARCH strategy (in this case for Embase). [file 13031_2023_534_MOESM1_ESM.docx]

**SEARCH strategy (in this case for Embase)**
1     research/ or analytical research/ or applied research/ or descriptive research/ or empirical research/ or evaluation research/ or mental health research/ or participatory research/ or population research/ or public health systems research/ or qualitative research.mp.
2     Reproducibility/ or Data Accuracy/
3     public policy/ or policy/ or health care policy/ or decision making/
4     exp "Peer Review"/
5     exp information dissemination/ or exp implementation science/
6     exp data integration/ or exp health care planning/
7     (practic* or action* or respons* or public health or decision* or polic*).mp.
8     empirical.mp.
9     ((operation* or action or applied or analytical or implement* or outcome or healthcare or empirical or peer
review*) adj3 research*).mp.
10     exp Information Dissemination/
11     (transfer* adj5 (knowledge or expertise or evidence)).mp.
**12     or/1-11**
13     evidence based practice/ or *methodology/ or *evidence based medicine/ or *evidence based nursing/ (117545)
14     (evidence adj5 (bas* or practic* or action* or respons* or public health or decision* or polic*)).mp.
**15     13 or 14**
16     exp "Warfare"/ or "exp War exposure"/
17     (war or wars or warfare or warzone*).mp.
18     (conflict adj5 (zone* or affect* or armed or area* or setting*)).mp.
19     humanitarian.mp.
20     exp Relief Work/
21     relief work.mp.
22     exp Refugees/
23     (refugee* or displaced person*).mp.
24     or/16-23
**25     12 and 15 and 24**

**Mesh Terms adaptations in the different Databases**

|  | ***Medline*** | ***Embase*** | ***Global Health*** |
| --- | --- | --- | --- |
| 1 | exp health services research/ or exp outcome assessment, health care/ or exp empirical research/ or exp operations research/ or exp peer review, research/ | research/ or analytical research/ or applied research/ or descriptive research/ or empirical research/ or evaluation research/ or mental health research/ or participatory research/ or population research/ or public health systems research/ or qualitative research | exp Research/ |
| 2 | "Reproducibility of Results"/ or Data Accuracy/ | Reproducibility/ or Data Accuracy/ | exp repeatability/ or exp monitoring/ |
| 3 | exp Policy Making/ or Public Policy/ | *public policy/ or *policy/ or *health care policy/ or *decision making/ | exp health policy/ or policy/ |
| 4 | exp "Peer Review"/ | exp peer review | exp evaluation/ |
| 5 | exp "diffusion of innovation"/ or exp implementation science | exp information dissemination/ or exp implementation science | exp communication/ |
| 6 | health plan implementation/ or health priorities/ | exp data integration/ or exp health care planning | exp project implementation/ |
| 10 | Exp information dissemination | Exp information dissemination |  |
| 13 | Exp Evidence-Based practice | evidence based practice/ or methodology/ or evidence based medicine/ or evidence based nursing/ | techniques/ or analytical methods/ or qualitative techniques/ or quantitative techniques/ or rapid methods/ or sampling/ |
| 16 | Exp “Warfare and armed conflicts” | Exp Warfare” or “war exposure” | exp war/ |
| 20 | Exp Relief Work | Exp Relief Work | exp emergency relief/ |
| 22 | Refugees | Exp Refugee | exp refugees/ |
